# Chimeric Element-Regulated MRI Reporter System for Mediation of Glioma Theranostics

**DOI:** 10.3390/cancers17142349

**Published:** 2025-07-15

**Authors:** Qian Hu, Jie Huang, Xiangmin Zhang, Haoru Wang, Xiaoying Ni, Huiru Zhu, Jinhua Cai

**Affiliations:** 1 Department of Radiology, Children’s Hospital of Chongqing Medical University, National Clinical Research Center for Child Health and Disorders, Ministry of Education Key Laboratory of Child Development and Disorders, No. 136, Zhongshan Second Road, Chongqing 400014, China; huqian960916@126.com (Q.H.); huangjie199501@163.com (J.H.); uncledarkness@163.com (X.Z.); wanghaoru615@126.com (H.W.); hrz201909@163.com (H.Z.); 2 Chongqing Engineering Research Center of Stem Cell Therapy, No. 136, Zhongshan Second Road, Chongqing 400014, China; 3 Department of Radiology, The Third Affiliated Hospital of Chongqing Medical University, Chongqing 401120, China; 652547@hostital.cqmu.edu.cn

**Keywords:** reporter gene, chimeric element-regulated, PEG3 promoter

## Abstract

**Background and Purpose:** Glioblastoma remains a therapeutic challenge with a poor prognosis despite multimodal treatments. Reporter-based magnetic resonance imaging (MRI) offers a promising approach for tumor visualization, but its efficacy depends on sufficient reporter gene expression. This study aimed to develop a chimeric element-regulated ferritin heavy chain 1 (*FTH1*) reporter system to enhance MRI-based glioma detection while enabling targeted therapy via transferrin receptor (TfR)-mediated drug delivery. **Methods:** Using gene cloning techniques, we constructed a chimeric *FTH1* expression system comprising tumor-specific *PEG3* promoter (transcriptional control), *bFGF-2 5′UTR* (translational enhancement), and *WPRE* (mRNA stabilization). Lentiviral vectors delivered constructs to U251 glioblastoma cells and xenografts. FTH1/TfR expression was validated by Western blot and immunofluorescence. Iron accumulation was assessed via Prussian blue staining and TEM. MRI evaluated T2 signal changes. Transferrin-modified doxorubicin liposomes (Tf-LPD) were characterized for size and drug loading and tested for cellular uptake and cytotoxicity in vitro. In vivo therapeutic efficacy was assessed in nude mouse models through tumor volume measurement, MR imaging, and histopathology. **Results:** The chimeric system increased *FTH1* expression significantly over PEG3-only controls (*p* < 0.01), with an increase of nearly 1.5-fold compared to the negative and blank groups and approximately a two-fold increase relative to the single promoter group, with corresponding TfR upregulation. Enhanced iron accumulation reduced T2 relaxation times significantly (*p* < 0.01), improving MR contrast. Tf-LPD (115 nm, 70% encapsulation) showed TfR-dependent uptake, inducing obvious apoptosis in high-TfR cells compared with that in controls. In vivo, Tf-LPD reduced tumor growth markedly in chimeric-system xenografts versus controls, with concurrent MR signal attenuation. **Conclusions:** The chimeric regulatory strategy overcomes limitations of single-element systems, demonstrating significant potential for integrated glioma theranostics. Its modular design may be adaptable to other reporter genes and malignancies.

## Simple Summary

This study developed an FTH1 expression system regulated by chimeric elements, leveraging the mechanism whereby FTH1 overexpression induces intracellular iron deficiency to activate upregulation of transferrin receptor (TfR) expression. Through the specific binding between TfR and transferrin (Tf), the system enables targeted delivery of anti-tumor agents to glioma cells, achieving dual functions of MRI-based glioma imaging and targeted therapy.

## 1. Introduction

Gliomas represent the most prevalent and aggressive primary brain tumors within the central nervous system [1]. Despite the application of comprehensive treatment such as surgical resection, radiotherapy, and chemotherapy, which can improve the prognosis of glioma, the median survival period remains only about 14 months [2,3]. The tumor’s high invasiveness and tendency to recur pose significant clinical challenges for early diagnosis and treatment.

Magnetic resonance imaging (MRI) has become a crucial clinical diagnostic tool due to its advantages of being radiation-free, non-invasive, and providing excellent soft-tissue contrast along with multi-sequence imaging capabilities. In recent years, MRI-based molecular imaging has opened new avenues for glioma diagnosis. This technology integrates MRI with molecular biology techniques, enabling non-invasive observation of pathophysiological changes at the cellular or molecular level in living tissues [4,5]. With the rapid advancement of molecular and cellular imaging technologies, it can now dynamically monitor cellular immunotherapy, including non-invasive tracking of cell localization, activation, and fate [6]. MRI reporter gene imaging is one of the key components of molecular imaging. *Ferritin heavy chain 1 (FTH1)*, as an MRI reporter gene, has been extensively validated for its imaging efficacy in experimental studies [7,8,9,10]. *FTH1* not only exhibits low cytotoxicity but also enables stable imaging through sustained expression [11]. Our previous research has shown that *FTH1* expression can induce intracellular iron accumulation detectable by MRI [12,13]; however, the expression levels of *FTH1* and the resulting MR imaging effects have not been optimal. Therefore, it is necessary to genetically modify or engineer *FTH1* to enhance its potential as a reporter gene for MR imaging.

We selected three types of gene regulatory elements to modify the *FTH1* gene. *Progression Elevated Gene-3 (PEG3)* is a broad-spectrum tumor-specific gene that is significantly overexpressed in human cancer cell lines while exhibiting minimal expression in normal tissue cells. In tumor cells, the activity of the *PEG3* promoter is regulated by the transcription factors *PEA3* and *AP-1*, leading to enhanced expression that specifically modulates the translation of downstream target genes within the tumor microenvironment [14]. The 5′ UTR of *basic fibroblast growth factor-2 (bFGF-2)* contains various higher-order structures, with a GC-rich segment that is unraveled in tumor cells due to the overexpression of translation initiation factor 4E (eIF4E), resulting in increased translation of downstream genes [15]. The woodchuck hepatitis virus post-transcriptional regulatory element (*WPRE*) is a potent RNA cis-acting element that regulates post-transcriptional levels by enhancing RNA polyadenylation. Inserting *WPRE* into the 3′ UTR of the target gene can significantly boost the expression rate of exogenous genes and prolong their expression duration [16]. The construction of a chimeric expression system using these components could potentially upregulate *FTH1* expression and its iron-accumulation capacity, thereby improving the detection sensitivity of MRI-based reporter gene imaging.

Tf, TfR, and *FTH1* play distinct roles in iron metabolism, collectively maintaining systemic iron homeostasis. Tf is responsible for iron transport, facilitating the transfer of ferric ions from serum to cells through specific binding to TfR on the cell membrane, followed by endocytosis and storage within *FTH1*. Research indicates that *FTH1*, as an iron storage protein, when overexpressed, can induce a deficiency of free iron within the cell [17], leading to an upregulation of TfR expression on the cell membrane and an increase in iron uptake. Based on this mechanism, *FTH1* can serve not only as an MRI reporter gene but also facilitate targeted drug delivery via the Tf-TfR transport system.

Liposomes (LP) are extensively studied drug delivery vehicles that, due to their favorable affinity for cell membranes, hollow structure, biocompatibility, stability, ease of modification, and cost-effectiveness, have emerged as an ideal choice for anticancer drug delivery systems [18,19]. Doxorubicin (DOX), a commonly used broad-spectrum anticancer agent, demonstrates significant efficacy; however, its clinical application is limited by severe adverse effects such as cardiotoxicity and myelosuppression [20,21,22]. Therefore, encapsulating DOX within LP carriers (LP-DOX, LPD) to enable tumor-targeted accumulation while minimizing off-target toxicity holds substantial clinical significance. As a nanomedicine delivery system, liposomes often utilize the enhanced permeability and retention (EPR) effect to achieve passive targeting, as seen with PEGylated liposomal doxorubicin (Doxil). Transferrin receptor (TfR)-targeted liposomes offer greater specificity, enabling more precise drug delivery to glioma cells expressing TfR and reducing drug distribution to normal tissues. While EPR-based passive delivery can also promote drug accumulation at tumor sites, it lacks specific targeting, which may result in some degree of drug exposure to healthy tissues [23].

Based on the aforementioned context, this study proposes to construct an *FTH1* expression system regulated by a chimeric element composed of the tumor-specific promoter *PEG3, bFGF-2 5′ UTR,* and *WPRE* *(PEG3-bFGF2 5′UTR-FTH1-WPRE)*. Through in vitro and in vivo experiments, we aim to validate the enhancing effect of this chimeric element on the expression of the MRI reporter gene *FTH1*. Simultaneously, leveraging the mechanism by which *FTH1* overexpression induces intracellular iron depletion to activate TfR upregulation, we will utilize the specific binding of TfR and Tf to deliver Tf-modified LPD (Tf-LPD) to glioma cells. This dual-function system is designed to achieve both MRI-based glioma monitoring and targeted therapy, thereby providing a novel integrated diagnostic and therapeutic strategy for glioma management.

## 2. Materials and Methods

### 2.1. Cell Preparation

The U251 human glioblastoma cell line was obtained from the Chongqing Key Laboratory of Child Neurodevelopment and Cognitive Disorders (Chongqing, China). Cells were cultured in Dulbecco’s Modified Eagle Medium (Gibco, Brooklyn, NY, USA) supplemented with 10% FBS (Gibco, USA) and 1% penicillin-streptomycin (Gibco, USA) in a 37 °C incubator with 5% CO_2_. Cells were subcultured regularly using trypsin/EDTA (Meilune, Dalian, China).

### 2.2. Mice

BALB/C nude female mice aged 4–5 weeks had 16 ± 2 g weights and were obtained from the Animal Experiment Center of Chongqing Medical University. Animal experiments in this study were approved by the Animal Ethics Committee of the Children’s Hospital of Chongqing Medical University, China, on 11 January 2024 (approval No. CHCMU-IACUC20240111010). The experimental protocols and procedures were conducted in accordance with the Guide for the Care and Use of Laboratory Animals (8th ed) (National Institutes of Health, 2011).

### 2.3. Plasmids and Lentivirus

The FTH1 reporter gene, PEG3 promoter, bFGF-2 5′UTR, WPRE, and the lentiviral vector packaging system were all constructed by GeneChem (Shanghai, China).

### 2.4. Main Reagents

Fetal bovine serum and DMEM culture medium were obtained from Gibco. Ferric ammonium citrate (FAC) was purchased from Sigma (Saint Louis, MO, USA). DSPE-PEG2000-Tf was acquired from Xi’an Ruixi Biological Technology Co., Ltd. (Xi’an, China). Ferritin Heavy Chain Rabbit pAb, GAPDH Mouse mAb (HRP), and Goat Anti-Rabbit IgG H&L (HRP) were all purchased from Zoonbio Biotechnology Co., Ltd. (Nanjing, China).

### 2.5. Preparation of Recombinant Lentiviral Vector

The *FTH1* reporter gene was ligated to the *PEG3* promoter, *bFGF-2 5′ UTR*, and *WPRE* through restriction enzyme digestion and amplification, resulting in the construction of two lentiviral plasmids: *U6-PEG3-FTH1-CMV-EGFP-EF1A-Luciferase-sv40-puromycin* and *U6-PEG3-bFGF2 5′UTR-FTH1-WPRE-CMV-EGFP-EF1A-Luciferase-sv40-puromycin*.Following sequencing validation, these lentiviral plasmids were co-transfected with the packaging plasmids *pSPAX2* and *pMD2G* into 293T cells. Viral supernatants were collected at 48 and 72 h post-transfection, and the resulting viral particles were concentrated via ultracentrifugation, designated as PEG3-FTH1-LV and PEG3-bFGF2 5′UTR-FTH1-WPRE-LV, respectively. The lentiviral particles were aliquoted and stored at −80 °C. A negative control group was established using the NC-LV, while U251 cells that were not subjected to lentiviral transfection served as the blank control group.

### 2.6. Lentiviral Transduction

U251 cells in the logarithmic growth phase were seeded at a density of 1.0 × 10^5^ cells per well in a 12-well plate and cultured in complete medium for 24 h at 37 °C with 5% CO_2_ to allow for adherence. The medium was aspirated, and the cells were washed three times with PBS. The complete medium was replaced, and the required lentiviral volume was calculated at an MOI of 5. HitransG P transduction enhancer was added according to the manufacturer’s instructions, and the cells were incubated. After 12–16 h, the supernatant was removed and replaced with complete medium, followed by further incubation. The expression of green fluorescent protein was observed under a fluorescence microscope 48 h post-infection.

### 2.7. Western Blot

Following the collection of untreated U251 cells and U251/NC, U251/PEG3-FTH1, and U251/PEG3-bFGF2 5′UTR-FTH1-WPRE cells transduced with lentivirus, protein was extracted. Protein concentration was determined via BCA assay. Following SDS-PAGE electrophoresis, proteins were transferred to PVDF membranes. Membranes were blocked with 5% non-fat milk for 1 h, followed by overnight incubation at 4 °C with primary antibodies against GAPDH (1:10,000) (Zen BioScience, Chengdu, Sichuan, China; Cat.R23306), FTH1 (1:2000) (Zen BioScience, Chengdu, Sichuan, China; Cat. 200350-F12), and TfR (1:1000) (Zen BioScience, Chengdu, Sichuan, China; R381603). Membranes were then incubated with secondary antibodies (1:3000) (Zen BioScience, Chengdu, Sichuan, China; Cat.511203) for 1–2 h and visualized using ECL reagent.

### 2.8. Immunofluorescence Assay

Following logarithmic growth, U251 cells from each group were harvested and seeded into confocal dishes for 24 h. Following PBS washes, cells were fixed with 4% paraformaldehyde (PFA, Beyotime, p0099, Shanghai, China) for 20 min. Post-fixation, cells were blocked with 5% normal goat serum at 37 °C for 1 h. To validate TfR overexpression, cells were incubated overnight at 4 °C with rabbit anti-TfR monoclonal antibody (1:250), followed by 1 h incubation with DyLight 488-conjugated goat anti-rabbit secondary antibody (1:500). Nuclei were counterstained with DAPI (1:1000). Visualization was performed using a laser confocal microscope (Nikon A1R, Shinagawa, Japan) at 600× magnification.

### 2.9. Prussian Blue Staining and Transmission Electron Microscopy

U251 cells from each group were cultured in complete medium supplemented with ferric ammonium citrate (FAC; Sigma-Aldrich, St. Louis, MO, USA) solution at a final concentration of 0.2 mmol/L. Following 72 h of incubation at 37 °C in a 5% CO_2_ incubator, the cells were washed three times with PBS. Subsequently, the cells were stained using a Prussian blue staining kit (nuclear fixation method), and the staining characteristics of the cells in each group were observed under a microscope.

U251 cells from each group were cultured in complete medium supplemented with 0.2 mmol/L FAC solution for 72 h at 37 °C in a 5% CO_2_ incubator. Following three washes with PBS, the supernatant was discarded after centrifugation. Cells were then fixed with 2.5% glutaraldehyde and stored at 4 °C. Samples were prepared, stained, dehydrated, and embedded for transmission electron microscopy (TEM) analysis.

For quantification of the intracellular iron content, 1 × 10^6^ cells per group were centrifuged and dried at 110 °C overnight. The cells were then digested in 500 μL of a nitric acid-perchloric acid mixture (3:1) at 60 °C for 3 h. The iron concentration was measured using an atomic absorption spectrophotometer (Huaguang HG-960 2A, Shenyang, China). Each sample was measured 3 times. The concentration values are presented in units of pg/cell.

### 2.10. MR Imaging of Cells

A final concentration of 0.2 mmol/L ferric ammonium citrate solution (FAC) (Sigma, Kanagawa, Japan) was added to the complete culture medium of the cells. Following a 72-h incubation with 0.2 mmol/L FAC, U251 cells from each group were washed thrice with PBS. The cells were then collected and allowed to sediment naturally before undergoing T2WI scanning using a 3.0 T clinical MR imaging system (Philips Healthcare, Best, Hoofddorp, The Netherlands). The T2WI scanning parameters were as follows: TR = 2424 ms, TE = 100 ms, FOV = 100 mm × 100 mm, voxel size = 0.620 mm × 0.600 mm, imaging matrix = 160 × 150, signal-to-noise ratio = 1, with a scan duration of 1 min 56 s.

### 2.11. Preparation of Tf-LPD

Tf-modified liposomes (Tf-LP) were prepared via the thin-film dispersion method. Briefly, HSPC (6 mg), cholesterol (2 mg), and DSPE-PEG2000-Tf (2 mg) (Xi’an ruixi Biological Technology Co., Ltd., Xi’an, China) were dissolved in 3 mL of chloroform and evaporated under reduced pressure to form a thin film. The film was hydrated with ammonium sulfate solution and sonicated. Following sonication and extrusion through a polycarbonate membrane (200 nm pore size), the liposomes were dialyzed using a nano-dialysis device (polycarbonate membrane, 10 nm pore size) to obtain transferrin-modified liposomes (Tf-LP).

Doxorubicin (Innochem, SY034864, Beijing, China) aqueous solution was added, and the mixture was sonicated and incubated at 50 °C for 1 h. Subsequently, the mixture was dialyzed again using a nano-dialysis device (polycarbonate membrane, 10 nm pore size) to yield transferrin-modified doxorubicin liposomes (Tf-LPD).

### 2.12. Identification of Tf-LPD

TEM was employed to observe the morphology of each liposome formulation, including Tf-LP and Tf-LPD. Liposome size was analyzed using a Malvern particle size analyzer. Doxorubicin encapsulation efficiency was determined via ultrafiltration centrifugation. Briefly, 300 μL of the Tf-LPD solution was added to an ultrafiltration tube and centrifuged at 5000 rpm for 10 min. The filtrate was collected, diluted with methanol, and the amount of free drug (M1) in the filtrate was quantified. An equal volume of Tf-LPD solution was also treated with methanol to disrupt the liposomes, and the total doxorubicin amount (M0) was determined. Encapsulation efficiency was then calculated.

### 2.13. Cellular Uptake Assays of Tf-LP

U251 cells from each group were cultured in complete medium (1% double antibody, 10% fetal bovine serum). Cells in the logarithmic growth phase were digested and transferred to confocal dishes for 24 h, followed by the addition of DiI-labeled Tf-LP. After co-incubation for 1, 3, 6, and 12 h, the cells were washed three times with PBS to remove unbound liposomes and fixed with paraformaldehyde. Subsequently, the cells were stained with a nuclear dye, and the binding of cells and liposomes was observed under a laser confocal microscope at 600× magnification.

### 2.14. Cytotoxicity Assays of Tf-LPD

The cytotoxic effects of Tf-LPD loaded with drugs on U251 cells were assessed using the CCK8 assay. Six wells per group were utilized, with a total of four groups: U251 cells, U251/NC cells (following lentiviral transduction), U251/PEG3-FTH1 cells, and U251/PEG3-bFGF2 5′UTR-FTH1-WPRE cells. Cells were seeded in 96-well plates, and 24 h later, 0, 1, 2, 4, 8, and 16 uL of Tf-LPD solution (1 mg/mL) were added to each well. After a 6-h incubation, CCK-8 reagent was added. Absorbance at 450 nm was measured using a multi-function microplate reader, and cell viability was calculated.

### 2.15. Flow Cytometry

U251 cells from each group were seeded at a density of 1 × 106 cells per well in 6-well plates and incubated at 37 °C with 5% CO_2_ for 24 h. Following cell adherence, the culture medium was removed, and the cells were washed with PBS. Subsequently, the Tf-LPD solution at a concentration of 8 μg/mL was added, and incubation continued for 24 h. After PBS washing, trypsin digestion was performed. The PBS wash and digested cell suspension were combined, and cells were collected by centrifugation at 1000 rpm for 5 min. The cells were washed twice with PBS. The cells were resuspended in 500 μL of 1× binding buffer, and the solution was transferred to a 5 mL flow cytometry tube. Annexin-FITC (5 μL) (BioLegend, San Diego, CA, USA) and PI (5 μL) (BioLegend, San Diego, CA, USA) were added, and the cells were mixed and incubated in the dark at room temperature for 10 min. Analysis was performed within 1 h using flow cytometry.

### 2.16. Establishment of U251 Cell Grafts in Nude Mice

U251 cells were cultured at 37 °C and 5% CO_2_, and the cells were taken at the logarithmic growth stage to establish the nude mouse U251 subcutaneous graft tumor model. Twenty tumor-bearing nude mice were randomly divided into four groups: (1) U251; (2) U251/NC; (3) U251/PEG3-FTH1; and (4) U251/PEG3-bFGF2 5′UTR-FTH1-WPRE. Intratumoral viral transfections were performed when the tumors of hormonal nude mice grew to a size of approximately 150 mm^3^. The first group was a control group without any intervention, the second group was injected with NC-LV, the third group was injected with PEG3-FTH1-LV, and the fourth group was injected with PEG3-bFGF2 5′UTR-FTH1-WPRE-LV. Intratumoral injections were administered every three days using the same procedure, for a total of 3–5 injections. Forty-eight hours following the final lentiviral injection, 100 μL of the luciferase (Luc) substrate, luciferin, was administered via intraperitoneal injection. Subsequently, tumor site fluorescence intensity was assessed using an in vivo imaging system.

### 2.17. In Vivo Biodistribution of Tf-LPD

DiR-labeled Tf-LPD, at a concentration of 5 mg/mL, was dissolved in distilled water. Both the non-transduced tumor-bearing mice and the three groups of tumor-bearing mice that had undergone successful in vivo lentiviral transduction were administered 0.2 mL of DiR-labeled Tf-LPD via tail vein injection. Fluorescence intensity in various tissues was observed at different time points using an in vivo small animal imaging system.

### 2.18. Tumor Suppression via Tf-LPD

Following intravenous administration via the tail vein, the therapeutic efficacy of drug-loaded liposomes was assessed in tumor-bearing mice. The treatment groups comprised three cohorts of mice that underwent successful in vivo lentiviral transduction and a control group of non-transduced tumor-bearing mice. Drug administration was performed every three days for a total of five administrations, with a dosage of 4 mg/kg per injection. Tumor dimensions, specifically the longest diameter (D1) and the shortest diameter (D2), were measured using a digital caliper. Tumor volume (Vtumor) was calculated using the formula: Vtumor = 1/2 × (D1 × D22). Tumor growth inhibition by the drug-loaded liposomes in the model animals was evaluated by plotting tumor relative growth curves.

### 2.19. MR Scanning of Animals

After the plasmid was stably expressed in the tumor of nude mice, T2WI scans were performed on each group of nude mice using a 3.0 T clinical MR imager (Philips Healthcare, Best, The Netherlands). 48 h after plasmid transfection, each group of tumor-bearing nude mice was injected with 200 uL of Tf-LPD solution (at a concentration of 1 mg/mL) via tail vein. Tumor size and morphology were observed by magnetic resonance scanning (MRI) before and on days 1, 3, and 7 after the injection of Tf-LPD solution, and the parameters of T2WI scanning were as follows: TR = 2424 ms, TE = 100 ms, FOV = 100 mm × 100 mm, voxel = 0.62 mm × 0.6 mm, imaging matrix = 160 × 150, signal-to-noise (S/N) ratio = 1, scan time was 1 min 56 s; scan time was 1 min 56 s. 160 × 150, S/N = 1, scan time 1 min 56 s; transverse position: TR = 4414 ms, TE = 80 ms, FOV = 65 mm × 65 mm, voxel = 0.5 mm × 0.5 mm, imaging matrix = 132 × 127, S/N = 1, scan time 5 min 44 s. T2 mapping Scanning parameters were as follows: TR = 2000 ms, TE1 = 1, scan time 5 min 44 s. 2000 ms, TE1 = 13 ms, △TE = 13 ms, TE8 = 104 ms, FOV = 80 mm × 80 mm, voxel = 0.5 mm × 0.55 mm, imaging matrix = 160 × 145, signal-to-noise ratio = 1, and scanning time was 3 min 56 s.

### 2.20. In Vivo Pharmacodynamics of Tf-LPD

The nude mice in each group of tumor-bearing nude mice were put to death on the 14th day after Tf-LPD injection, and the tumors, hearts, lungs, livers, spleens, and kidneys were excised sequentially. Tumor size was collected from each group for evaluation. Excised tissue specimens were fixed in tissue fixative containing 4% paraformaldehyde to maintain their morphological and structural integrity. After fixation for an appropriate period of time (usually 24 h), the tissue specimens were embedded, and sections were prepared for subsequent immunohistochemistry, Prussian blue staining, and HE staining. Finally, images are captured using a microscope.

### 2.21. Statistical Analysis

Each test was repeated at least three times; the quantitative data are shown as means ± standard deviations (SDs). These data were analyzed using GraphPad Prism 5.01 (GraphPad Software, Inc., San Diego, CA, USA) and SPSS 17.0 statistical software (SPSS Inc., Chicago, IL, USA). The data were analyzed by paired t-test between two groups and one-way ANOVA for more than two groups, followed by Tukey’s post hoc test. *p* < 0.05 was considered statistically significant.

## 3. Results

### 3.1. Lentiviral Transduction

**Figure 1:**
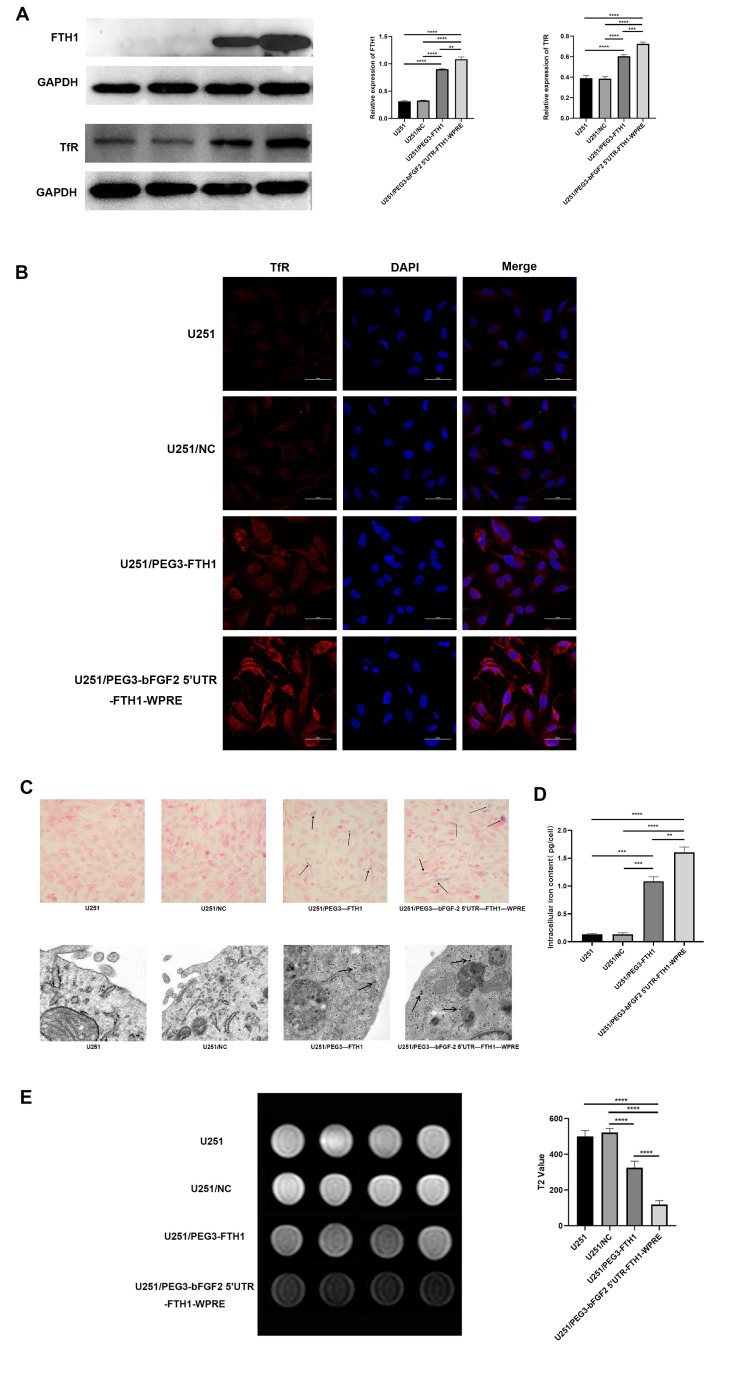
FTH1 and TfR expression, iron trafficking effects, and MR imaging ((**A**): After lentiviral transfection, differences in FTH1 and TfR protein expression were observed among the U251 cell groups (**** *p* < 0.0001,*** *p* < 0.001,** *p* < 0.01; (**B**): Immunofluorescence results indicated significant variations in surface TfR expression across the U251 cell groups; (**C**): The Prussian blue staining assay revealed blue-stained granules (indicated by arrows) within the U251/PEG3-FTH1 and U251/PEG3-bFGF2 5′UTR-FTH1-WPRE cell groups, while TEM showed black iron granules (indicated by arrows) in these same groups; (**D**): The iron content in the U251/PEG3-FTH1 group and the U251/PEG3-bFGF2 5′UTR-FTH1-WPRE group was significantly higher than that in the U251 group and the U251/NC group (**** *p* < 0.0001). No significant difference was observed between the U251 group and the U251/NC group. (**E**): Magnetic resonance imaging T2-weighted images and histogram analysis of grayscale values demonstrated notable differences in signal intensity among the U251 cell groups).

We successfully constructed recombinant lentiviral vector plasmids PEG3-FTH1-LV and PEG3-bFGF2 5′UTR-FTH1-WPRE-LV. Sequencing results confirmed the expected constructs. Following 48 h of lentiviral transduction of U251 cells, green fluorescent protein expression was observed via fluorescence microscopy (Figure 1A).

### 3.2. FTH1 and TfR Expression

Western blot analysis revealed negligible FTH1 expression in the U251 and U251/NC groups. Similarly, TfR expression remained at basal levels, showing no statistically significant intergroup difference (*p* > 0.05). In contrast, both the U251/PEG3-FTH1 and U251/PEG3-bFGF2 5′UTR-FTH1-WPRE groups exhibited significantly increased FTH1 and TfR expression compared to the former groups. Furthermore, the U251/PEG3-bFGF2 5′UTR-FTH1-WPRE group demonstrated higher expression levels than the U251/PEG3-FTH1 group (Figure 1A). These findings indicate that the PEG3 promoter and chimeric element regulate the overexpression of FTH1 and TfR in U251 cells, with the chimeric element demonstrating a more pronounced effect than the PEG3 promoter alone.

Immunofluorescence analysis revealed no significant difference in the intensity of red fluorescence on the cell surface between the U251 and U251/NC groups. However, the U251/PEG3-FTH1 and U251/PEG3-bFGF2 5′UTR-FTH1-WPRE groups exhibited a higher intensity of red fluorescence compared to the former two groups, with the U251/PEG3-bFGF2 5′UTR-FTH1-WPRE group displaying a more intense signal than the U251/PEG3-FTH1 group (Figure 1B). These immunofluorescence findings were consistent with the Western blot results, indicating that transduction with PEG3-FTH1-LV and PEG3-bFGF2 5′UTR-FTH1-WPRE-LV not only regulated intracellular FTH1 overexpression but also induced the upregulation of TfR on the cell surface, thereby providing a foundation for subsequent targeted therapy.

### 3.3. Iron Transfer Effect

Prussian blue staining revealed intracellular blue-stained iron particles in both the U251/PEG3-FTH1 and U251/PEG3-bFGF2 5′UTR-FTH1-WPRE groups, whereas no blue-stained iron particles were observed in the U251 and U251/PEG3-NC groups. TEM demonstrated the presence of black iron particles within the U251/PEG3-FTH1 and U251/PEG3-bFGF2 5′UTR-FTH1-WPRE groups, while no such particles were detected in the U251 and U251/PEG3-NC groups (Figure 1C).

Quantitative analysis of intracellular iron content (Figure 1D) revealed that the iron level in U251/PEG3-FTH1 cells was 1.11 ± 0.56 pg per cell, while the U251/PEG3-bFGF2 5′UTR-FTH1-WPRE group exhibited a significantly higher iron content of 1.60 ± 0.67 pg per cell. Both were markedly elevated compared to the U251 control group (0.13 ± 0.01 pg per cell) and the U251/NC group (0.13 ± 0.19 pg per cell) (*p* < 0.0001).

### 3.4. Cellular MRI

MRI scans revealed that the U251/PEG3-FTH1 and U251/PEG3-bFGF2 5′UTR-FTH1-WPRE groups exhibited significantly lower signal intensity on T2-weighted images compared to the U251 and U251/PEG3-NC groups, with the signal intensity of the U251/PEG3-bFGF2 5′UTR-FTH1-WPRE group being nearly half that of the U251/PEG3-FTH1 group (Figure 1E). This observation supports the hypothesis that FTH1 overexpression and TfR upregulation, under conditions of FAC availability, lead to intracellular iron accumulation, consequently resulting in reduced T2WI signal intensity.

### 3.5. Characterization of Tf-LPD

**Figure 2:**
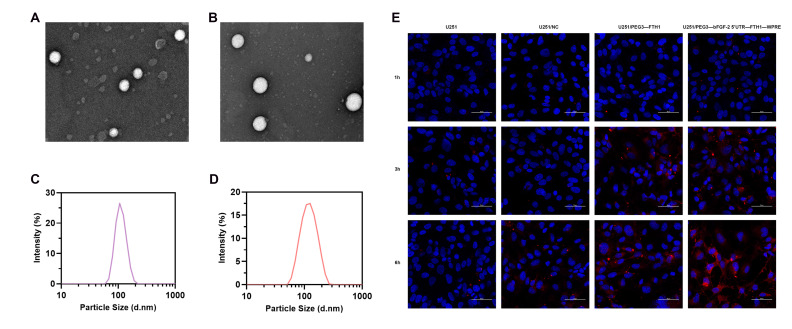
Characterization and in vitro uptake of Tf-LP and Tf-LPD ((**A**,**B**): TEM revealed that both Tf-LP and Tf-LPD exhibit spherical morphology and uniform size as nanomaterials; (**C**,**D**): Particle size analysis indicated that the diameter of Tf-LPD is (114.94 ± 3.17) nm, while Tf-LP has a diameter of (112.33 ± 6.53) nm; (**E**): Immunofluorescence results demonstrated that the uptake of Tf-LP by U251 cells increased over time, with the U251/PEG3-bFGF2 5′UTR-FTH1-WPRE group showing the highest uptake, Scale bar: 50 μm).

The synthesized liposomes exhibited a spherical morphology under transmission electron microscopy, with a uniform particle size distribution (Figure 2A,B). The average particle size of Tf-LPD was 114.94 ± 3.17 nanometers (Figure 2C), with a polydispersity index (PDI) of 0.202 ± 0.01 and a zeta potential of −15.78 ± 1.84 millivolts; in comparison, Tf-LP had an average particle size of 112.33 ± 6.53 nanometers (Figure 2D), a PDI of 0.254 ± 0.04, and a zeta potential of −8.08 ± 0.64 millivolts. The encapsulation efficiency of Tf-LPD was 70%, with a drug loading capacity of 7%. Liposomes with particle sizes smaller than 10 nm are rapidly cleared by the kidneys and have difficulty reaching the brain to exert their effects. Conversely, liposomes larger than 200 nm tend to accumulate in the liver and spleen, which hinders their ability to penetrate the blood-brain barrier. Therefore, we selected liposomes with an average particle size of approximately 100 nm for drug delivery.

### 3.6. Cellular Uptake of Tf-LP

Confocal microscopy analysis revealed differential uptake of DiI-labeled Tf-LP (red fluorescence) by U251 cells across the treatment groups (Figure 2E). Time-dependent increases in fluorescence intensity were observed in all U251 cell groups with extended co-incubation, indicating a time-dependent cellular uptake of the liposomes. Furthermore, no significant difference in fluorescence intensity was observed between the U251 and U251/NC groups. The U251/PEG3-FTH1 and U251/PEG3-bFGF2 5′UTR-FTH1-WPRE groups exhibited higher fluorescence intensity compared to the former two groups, with the U251/PEG3-bFGF2 5′UTR-FTH1-WPRE group displaying the most intense fluorescence. This observation may be attributed to the enhanced Tf-LP uptake in cells with increased TfR expression on the cell surface.

### 3.7. Cytotoxicity of Tf-LPD

**Figure 3:**
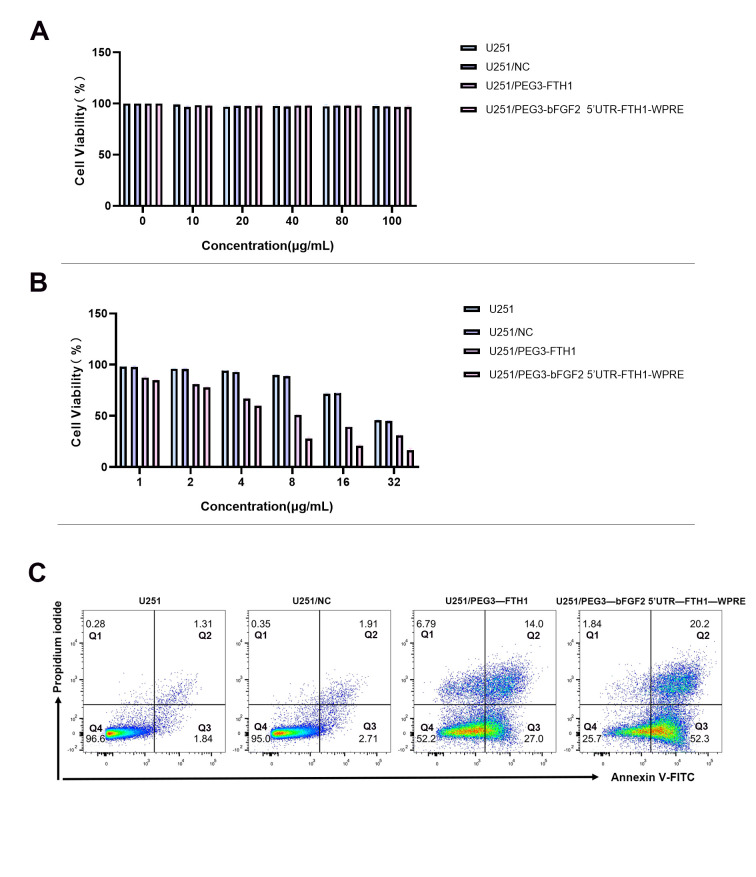
Cytotoxicity of Tf-LP and Tf-LPD ((**A**):The CCK8 assay results indicate that Tf-LP exhibits minimal cytotoxicity across all U251 cell groups; (**B**): The results of the CCK8 assay indicate that the survival rate of U251 cells in all groups decreased with increasing concentrations of Tf-LPD, with the U251/PEG3-bFGF2 5′UTR-FTH1-WPRE group exhibiting the lowest cell survival rate; (**C**): The results of the flow cytometry experiments indicated that the apoptosis rates for the four groups of cells—U251, U251/NC, U251/PEG3-FTH1, and U251/PEG3-bFGF2 5′UTR-FTH1-WPRE—were 3.15%, 4.62%, 41%, and 72.5%).

The CCK-8 assay revealed that the unloaded Tf-LP exhibited negligible cytotoxicity against all U251 cell groups, indicating that the prepared targeted liposomes, when used as drug carriers, possessed minimal inherent cytotoxicity (Figure 3A). Furthermore, we assessed the in vitro therapeutic efficacy of Tf-LPD via the CCK-8 assay (Figure 3B). No significant differences in the survival rates of each U251 cell group were observed with the addition of 0, 1, 2, and 4 uL of the Tf-LPD solution. However, at 8 uL and 16 uL of Tf-LPD solution, the survival rates of the U251/PEG3-FTH1 and U251/PEG3-bFGF2 5′UTR-FTH1-WPRE groups were significantly lower than those of the U251 and U251/PEG3-NC groups, with the U251/PEG3-bFGF2 5′UTR-FTH1-WPRE group exhibiting a lower survival rate than the U251/PEG3-FTH1 group. This suggests that Tf-LPD, at the same concentration and volume, demonstrated enhanced cytotoxicity against U251 cells with higher TfR expression.

Based on the CCK-8 assay results, the concentration of Tf-LPD was determined to be 8 μg/mL. Subsequently, apoptosis analysis was performed via flow cytometry, and the results are presented in Figure 3C. The apoptotic rate was quantified by summing the late and early apoptotic rates. The apoptotic rates for the U251, U251/NC, U251/PEG3-FTH1, and U251/PEG3-bFGF2 5′UTR-FTH1-WPRE groups were 3.15%, 4.62%, 41%, and 72.5%, respectively. These findings indicate that Tf-LPD, at the same concentration, exhibited a more pronounced cytotoxic effect on U251 cells, which displayed higher TfR expression on their cell surfaces.

### 3.8. Transfection in Vivo

**Figure 4:**
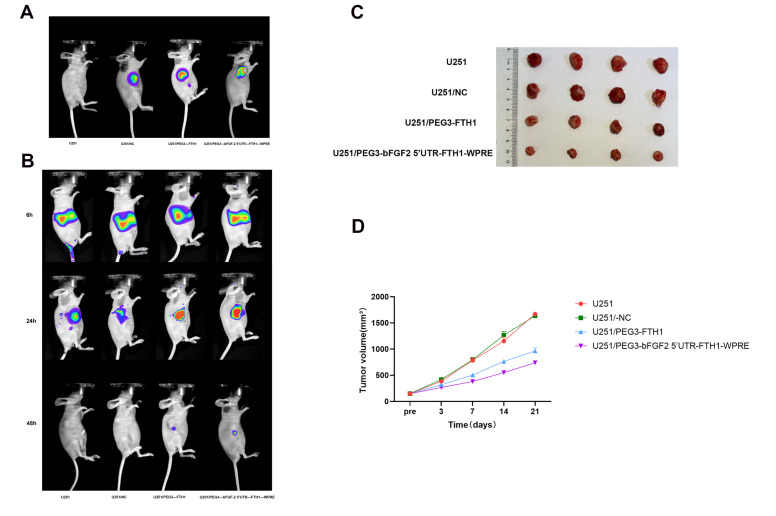
Fluorescent distribution maps of lentivirus and Tf-LPD in mice, growth curve of tumors ((**A**): Significant fluorescence distribution was observed in the tumor sites of mice from the three groups following in vivo transfection with lentiviral vectors; (**B**): After entering the nude mice via the tail vein, Tf-LPD primarily accumulates in the liver at 6 h, and at 24 h, it is predominantly found in the tumor. The fluorescence signals in the U251/PEG3-FTH1 and U251/PEG3-bFGF2 5′UTR-FTH1-WPRE groups are significantly stronger than those in the U251 and U251/-NC groups, with nearly complete metabolism observed by 48 h; (**C**): On day 21, the tumors from each group of mice were dissected, revealing that the U251/PEG3-bFGF2 5′UTR-FTH1-WPRE group exhibited the smallest tumor size; (**D**): By systematically measuring and recording tumor growth in various groups of mice, we generated growth curves. The results indicate that the tumor growth rates in the U251 and U251/-NC groups showed no significant differences and were faster than those in the other two groups, while the U251/PEG3-bFGF2 5′UTR-FTH1-WPRE group exhibited the slowest tumor growth rate).

Following the successful establishment of a U251 subcutaneous xenograft model in nude mice, intratumoral viral transduction was performed. At 48 h post-final transduction, in vivo fluorescence microscopy was employed to assess the transduction efficiency. Significant fluorescent signals were observed within the tumor sites of the U251/NC, U251/PEG3-FTH1, and U251/PEG3-bFGF2 5′UTR-FTH1-WPRE groups (Figure 4A), indicating successful intratumoral viral transduction and validating the progression to subsequent experiments.

### 3.9. In Vivo Biodistribution of Tf-LPD

Following intravenous injection of DiR-labeled Tf-LPD into tumor-bearing mice, in vivo fluorescence imaging revealed the results shown in Figure 4B. Six hours post-injection, the liposomes primarily accumulated in the liver. After 24 h, they were observed to have aggregated within the tumor, with the U251/PEG3-FTH1 and U251/PEG3-bFGF2 5′UTR-FTH1-WPRE groups exhibiting significantly stronger fluorescence signals compared to the U251 and U251/-NC groups. By 48 h, the liposomes were largely metabolized. This indicates that transferrin-modified liposomes enhance the targeting capability towards cells with elevated TfR expression, which is consistent with the cellular uptake findings.

### 3.10. Anti-Tumor Effect of Tf-LPD

Tumor volumes in U251, U251/-NC, U251/PEG3-FTH1, and U251/PEG3-bFGF2 5′UTR-FTH1-WPRE xenograft models were monitored over 21 days. Tumor volumes were dynamically measured using calipers prior to the initial intravenous injection of the drug-loaded liposomes and on days 3, 7, 14, and 21 post-injection. The results, shown in Figure 4C,D, indicated no significant difference in tumor size between the U251 and U251/-NC groups on day 21. However, these two groups exhibited tumor sizes nearly double those of the U251/PEG3-FTH1 group and approximately 1.5 times larger than the U251/PEG3-bFGF2 5′UTR-FTH1-WPRE group. These findings indicate that the drug-loaded liposome Tf-LPD demonstrated the most effective targeted inhibition of U251/PEG3-bFGF2 5′UTR-FTH1-WPRE tumors.

### 3.11. MR Scanning of Animals

**Figure 5:**
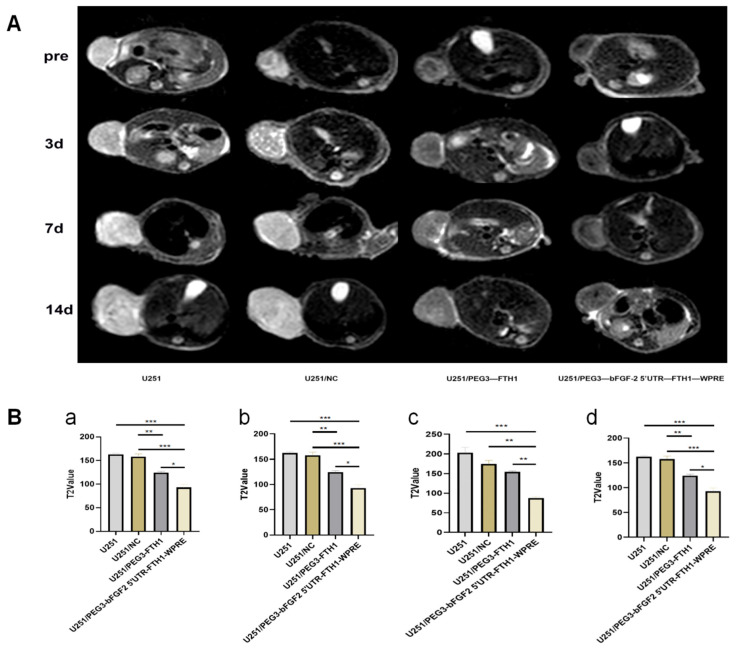
(**A**): Axial T2-weighted imaging of tumors in each group at various time points; (**B**): Analysis of T2 values for tumors in each group at different time intervals (a, b, c, and d represent the T2 signal intensity measurements obtained before and at 3, 7, and 14 days after intravenous administration of drug-loaded liposomes in tumor-bearing mice, respectively). Analysis of the T2WI scans and T2 grayscale values revealed that the U251 and U251/NC groups exhibited no significant differences in tumor signal intensity, both showing slightly elevated signals. In contrast, the U251/PEG3-FTH1 group displayed a lower tumor signal compared to the first two groups, while the U251/PEG3-bFGF2 5′UTR-FTH1-WPRE group demonstrated a more pronounced reduction in tumor signal intensity) (*** *p* < 0.001,** *p* < 0.01,* *p* <0.05).

Following intravenous administration of drug-loaded liposomes, MRI scans were performed on the tumor-bearing mice at days 3, 7, and 14. Analysis of T2WI signals revealed no significant difference in signal intensity within the tumor regions of the U251 and U251/NC groups, both exhibiting slightly elevated signals. The U251/PEG3-FTH1 group displayed lower signal intensity compared to the aforementioned groups, while the U251/PEG3-bFGF2 5′UTR-FTH1-WPRE group exhibited a more pronounced reduction in tumor signal intensity (Figure 5). These findings are consistent with the cellular MRI results, indicating that infection with PEG3-FTH1-LV and PEG3-bFGF2 5′UTR-FTH1-WPRE-LV led to increased iron accumulation within the tumors, resulting in lower T2WI signals.

### 3.12. Pathologic Examination

**Figure 6:**
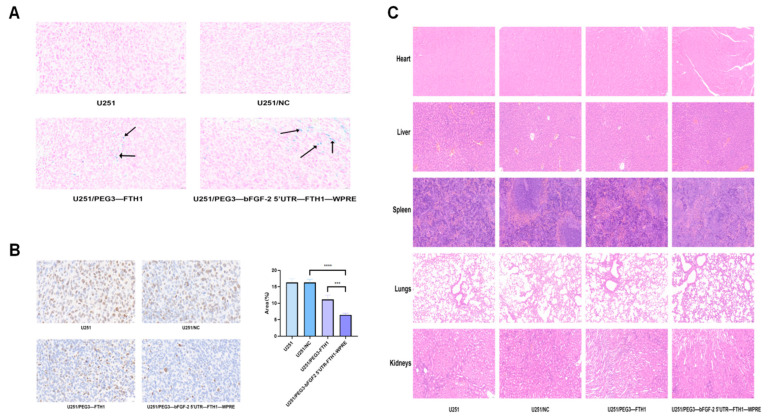
Immunohistochemistry, Prussian blue staining of tumor tissue, and HE staining of major organs ((**A**): Both the U251/PEG3-FTH1 group and the U251/PEG3-bFGF2 5′ UTR-FTH1-WPRE group exhibited blue-stained granules (indicated by arrows) within the tumor tissue cells, Scale bar: 20 μm; (**B**): The analysis of the proportion of Ki-67 immunohistochemically positive cells in tumor tissues revealed that the positive cell area percentages for the four groups were 16.34 ± 0.9, 16.29 ± 1.17, 11.15 ± 1.47, and 6.47 ± 0.56, (*** *p* < 0.001,**** *p* < 0.0001), Scale bar: 20 μm; (**C**): HE staining results indicate that the primary immune organ cell structures in all four groups of mice showed no significant damage, Scale bar: 50 μm).

Prussian blue staining of tumor sections from each group of mice revealed no discernible blue-stained particles within the U251 and U251/NC groups. Conversely, blue-stained particles were observed in the tumor tissues of the U251/PEG3-FTH1 and U251/PEG3-bFGF2 5′UTR-FTH1-WPRE groups (Figure 6A).

Immunohistochemical analysis of Ki-67 expression in tumor tissues revealed the following percentages of positively stained cells: 16.34 ± 0.9 for the U251 group, 16.29 ± 1.17 for the U251/NC group, 11.15 ± 1.47 for the U251/PEG3-FTH1 group, and 6.47 ± 0.56 for the U251/PEG3-bFGF2 5′UTR-FTH1-WPRE group (Figure 6B). The percentage of Ki-67-positive cells was significantly lower in the U251/PEG3-FTH1 and U251/PEG3-bFGF2 5′UTR-FTH1-WPRE groups compared to the U251 and U251/NC groups, with the lowest percentage observed in the U251/PEG3-bFGF2 5′UTR-FTH1-WPRE group. These findings suggest enhanced therapeutic efficacy of the drug-loaded liposomes in the U251/PEG3-FTH1 and U251/PEG3-bFGF2 5′UTR-FTH1-WPRE groups compared to the U251/PEG3-FTH1 group; the U251/PEG3-bFGF2 5′UTR-FTH1-WPRE group exhibited a slightly stronger tumor-suppressive effect.

Histopathological analysis of the heart, liver, spleen, lung, and kidney tissues from each group of mice, as revealed by HE staining, is presented in Figure 6C. No significant pathological damage was observed in the major immune organ cell structures across all groups. These findings suggest that neither the drug treatments nor the MRI scans induced adverse effects in the animals, indicating favorable biosafety profiles.

## 4. Discussion

This study successfully established an *FTH1* reporter gene expression system regulated by a chimeric element composed of the *PEG3* promoter, *bFGF-2 5′ UTR*, and *WPRE*. Both in vitro and in vivo experiments confirmed that this chimeric component significantly enhances the specific expression of the MRI reporter gene *FTH1* in gliomas. Furthermore, we integrated the FTH1-mediated TfR upregulation mechanism with Tf-modified drug-loaded liposomes (Tf-LPD) for targeted therapy, achieving a unified diagnostic and therapeutic approach for gliomas. The innovation of this strategy lies in: (1) addressing the insufficient expression efficiency of FTH1 through synergistic regulation of multiple components while ensuring specificity of FTH1 expression within tumors and (2) utilizing the bidirectional regulatory mechanism of FTH1-TfR to simultaneously enhance MRI imaging sensitivity and drug targeting. This research enables FTH1-mediated tumor MRI visualization and targeted therapy, providing a multimodal approach for gliomas, which also holds significant reference value for the treatment of pancreatic cancer, breast cancer, and other related malignancies.

The efficacy of MRI reporter imaging is determined by the expression levels of the reporter gene within cells or tissues. This study utilized a chimeric construct comprising the *PEG3* promoter, *bFGF-2 5′ UTR*, and *WPRE* to achieve specific and high expression of the *FTH1* reporter gene. Compared with the single *PEG3* promoter, the chimeric construct not only ensured specificity of *FTH1* expression in tumor tissues but also significantly enhanced the expression efficiency of *FTH1* at different levels. Firstly, the *PEG3* promoter is activated specifically in tumor cells by the *PEA3/AP-1* transcription factors, regulating the specific expression of *FTH1* at the transcriptional level, thereby ensuring *FTH1* expression is restricted to tumor tissues and absent in normal tissues. Secondly, the *bFGF-2 5′ UTR*, rich in “GC” content and highly structured, may participate in the translational control of numerous mRNAs. In normal cells, these structures inhibit the translation of downstream genes; however, in tumor cells, these structures are unwound by the translation initiation factor *eIF4E*, losing their original function of blocking downstream gene translation, resulting in the overexpression of downstream genes [24,25,26]. Additionally, *WPRE* may enhance the sustained expression of the protein by prolonging the mRNA half-life and promoting polyadenylation, further augmenting *FTH1* expression at the post-transcriptional level [27]. This chimeric system may not be limited to the *FTH1* reporter gene; it could also be applicable to other reporter genes, such as luciferase used in optical imaging.

The Tf-TfR transport system holds dual implications: (1) Imaging aspect: Increased iron uptake mediated by TfR can enhance the magnetic susceptibility and imaging effects of FTH1. In this study, both in vivo and in vitro MR imaging demonstrated that the chimeric element regulation group exhibited a more pronounced reduction in T2-weighted imaging (T2WI) signals compared to the PEG3 promoter group. (2) Therapeutic aspect: The upregulation of TfR provides a foundation for Tf-mediated targeted drug delivery. Flow cytometry in vitro revealed a significant increase in Tf-LPD uptake in the chimeric element group compared to the control group. Both in vivo and in vitro experiments indicated that the cytotoxic effect of Tf-LPD on gliomas in the chimeric element group was markedly greater than that of the control group. These results suggest that the upregulation of TfR induced by *FTH1* overexpression can significantly enhance the efficiency of targeted drug delivery.

In vivo gene delivery is pivotal for gene therapy and diagnostics, utilizing two primary methodologies: viral and non-viral vectors, each characterized by unique mechanisms, benefits, and drawbacks. Non-viral vectors predominantly consist of lipid nanoparticles (LNPs) and extracellular vesicles (EVs). LNPs are extensively researched for their capacity to encapsulate and safeguard genes, thereby enhancing delivery efficacy. They exhibit favorable biocompatibility, yielding non-toxic degradation products. However, their limited aqueous solubility may lead to drug leakage, and their larger size complicates targeting despite surface modifications [28,29,30]. EVs, currently a focal point of research, provide excellent biocompatibility, low immunogenicity, the ability to penetrate the blood-brain barrier, and versatile cargo loading capabilities (e.g., drugs, proteins, mRNA). Nonetheless, challenges in production, low yields, and potential instability hinder their large-scale application [31,32]. Viral vectors encompass adenoviruses, adeno-associated viruses (AAVs), and lentiviruses. Adenoviruses are straightforward to prepare and can infect a variety of cell types but elicit robust immune responses and result in transient gene expression [33,34]. AAVs circumvent genomic integration, thereby minimizing oncogenic risks, yet their limited genome size restricts insert capacity, and exogenous gene expression is often delayed [35,36]. Lentiviruses integrate stably into the host genome, facilitating long-term expression and accommodating larger inserts without generating infectious particles [37]. Whether in the preclinical research phase or during clinical trials, both the FDA and EMA impose stringent requirements on the production and use of lentiviral vectors. We opted for lentiviruses due to their sustained expression and high safety profile. In this study, we implemented a strategy involving multiple intratumoral injections of lentivirus in vivo, which offers the advantages of direct vector delivery to circumvent the blood-brain barrier limitations, enhancing transfection uniformity through multi-point distribution, and mitigating systemic toxicity risks with localized high titers. In vivo fluorescence imaging revealed significant fluorescence in the tumors of all three groups of tumor-bearing mice transfected with lentivirus, indicating successful in vivo transfection.

## 5. Conclusions

In summary, this study successfully integrated a chimeric element composed of the *PEG3* promoter, *bFGF-2 5′ UTR*, and *WPRE* with a magnetic resonance imaging (MRI) reporter gene, establishing a chimeric element-regulated *FTH1* expression system. In vitro and in vivo experiments validated that the chimeric element significantly enhances the specific expression of the MRI reporter gene *FTH1* in gliomas, thereby achieving improved contrast imaging on MR T2-weighted images. Additionally, based on the upregulation of cell surface transferrin receptors (TfR) due to iron deficiency mechanisms, we successfully developed uniform Tf-modified doxorubicin liposomes (Tf-LPD). By leveraging the specific binding of TfR and transferrin (Tf), Tf-LPD was targeted to glioma cells, facilitating dual functions of MRI imaging monitoring and targeted therapy for gliomas, thus providing a novel theranostics strategy for glioma management.

This study has certain limitations. Firstly, we employed intratumoral multiple-point injection of lentivirus for in vivo transfection; however, the absence of a control group using alternative transfection methods precludes the assessment of transfection efficiency. Future studies could compare the transfection efficiency between tail vein injection of lentivirus and multiple intratumoral injections of lentivirus. Secondly, this study utilized a subcutaneous animal model to specifically investigate whether the designed chimeric components could induce overexpression of the reporter gene in tumor cells and enhance targeted uptake of anticancer drugs. However, there are notable differences between the subcutaneous glioma model and the orthotopic model, such as variations in the tumor microenvironment and the ability of drugs to cross the blood-brain barrier. Future research will involve the use of orthotopic modeling to address these issues more comprehensively. Additionally, the drug-loaded liposomes Tf-LPD developed in this study are solely intended to meet the requirements for targeted delivery; subsequent studies could focus on the development of nanocarrier materials with greater technical complexity.

## Data Availability

The original contributions presented in this study are included in the article/Supplementary Materials. Further inquiries can be directed to the corresponding author.

## Supplementary Materials

The following supporting information can be downloaded at: https://www.mdpi.com/article/10.3390/cancers17142349/s1.
