# Supplementary figures and images for: Chimeric Element-Regulated MRI Reporter System for Mediation of Glioma Theranostics
Source: Cancers (Basel). 2025 Jul 15;17(14):2349. doi: 10.3390/cancers17142349 (PMC12293648; doi:10.3390/cancers17142349)

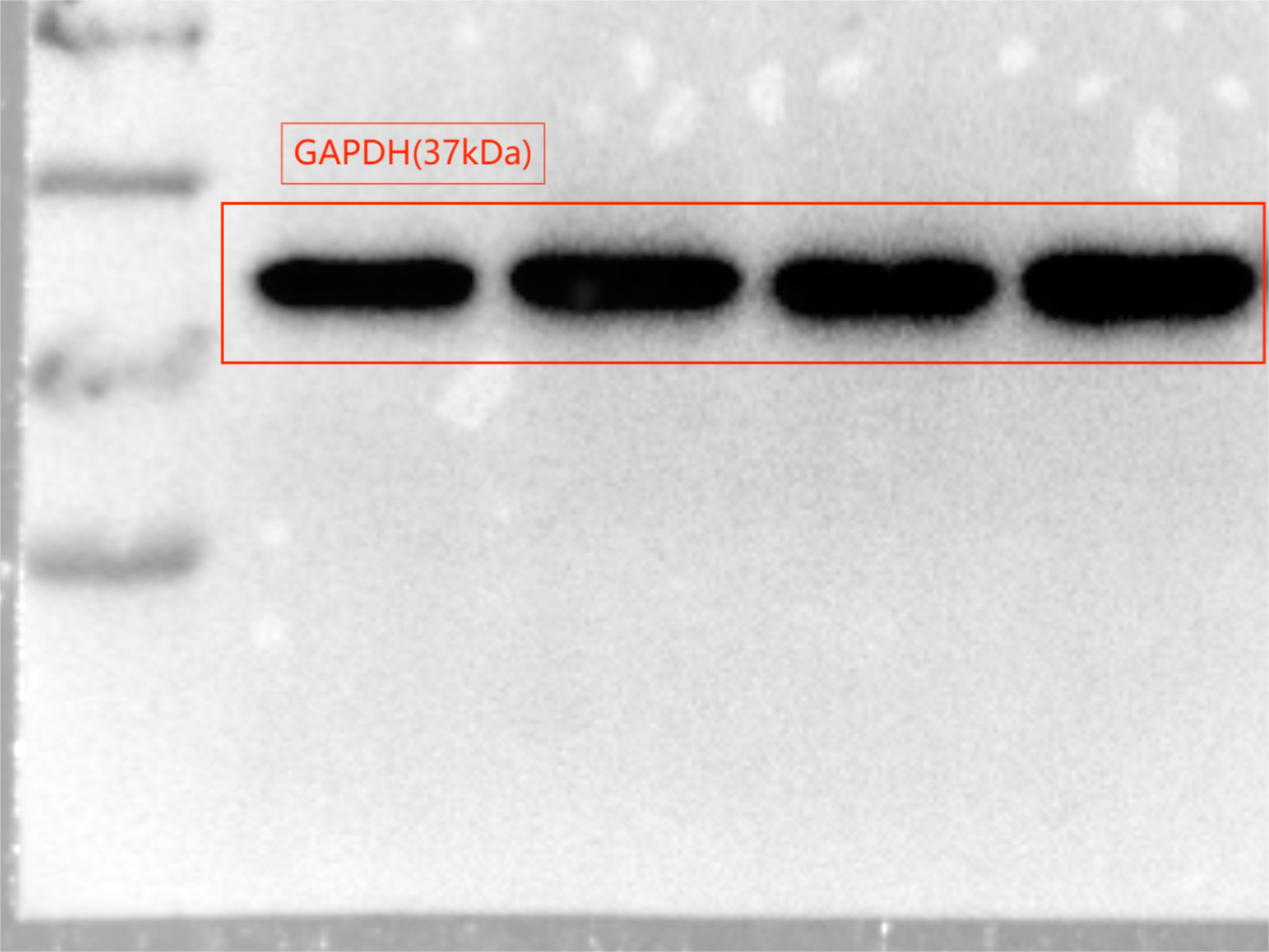

Supplement: Supplementary file 1 [file cancers-17-02349-s001.zip › WB/FTH1-GAPDH.png]

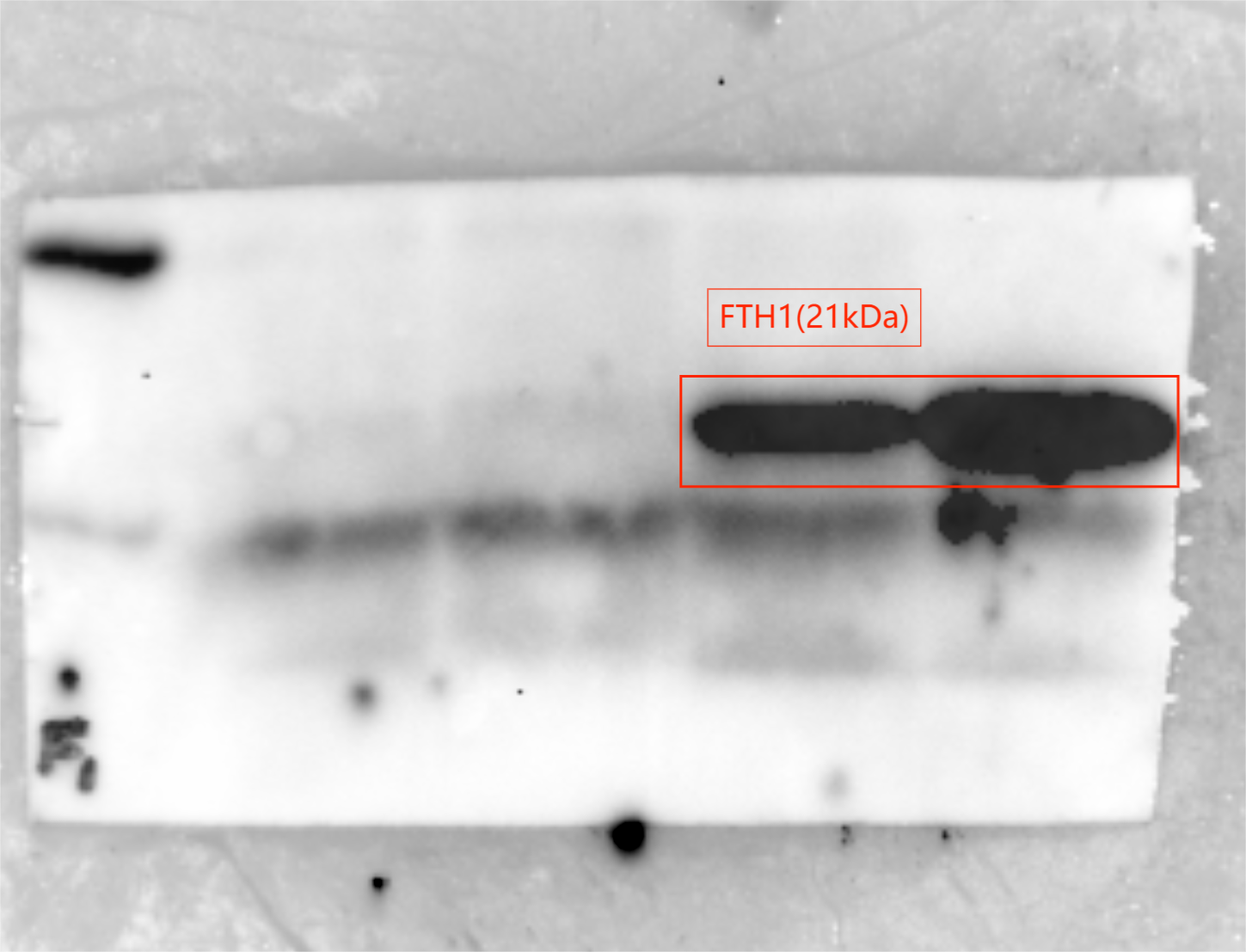

Supplement: Supplementary file 1 [file cancers-17-02349-s001.zip › WB/FTH1.png]

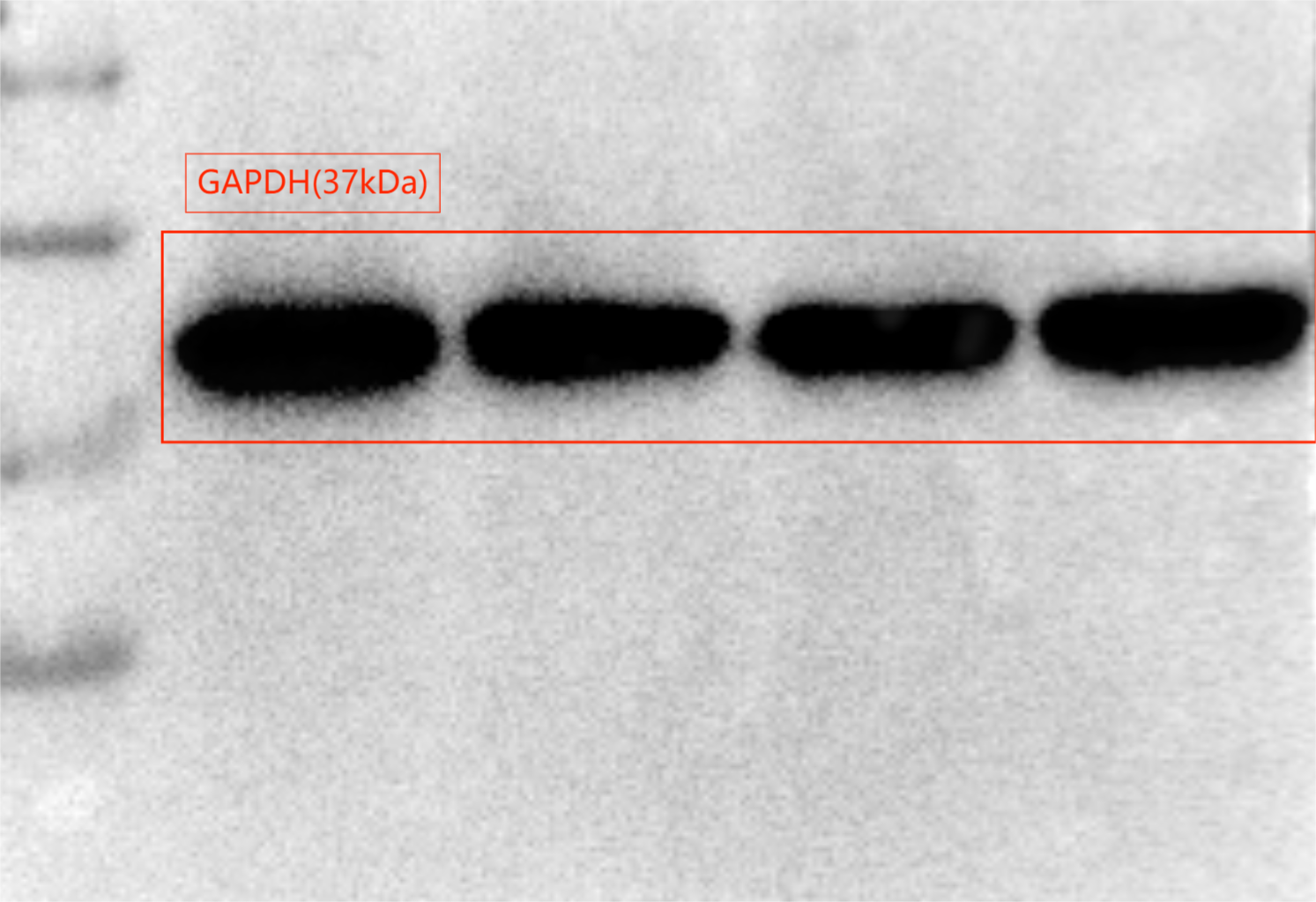

Supplement: Supplementary file 1 [file cancers-17-02349-s001.zip › WB/TfR-GAPDH.png]

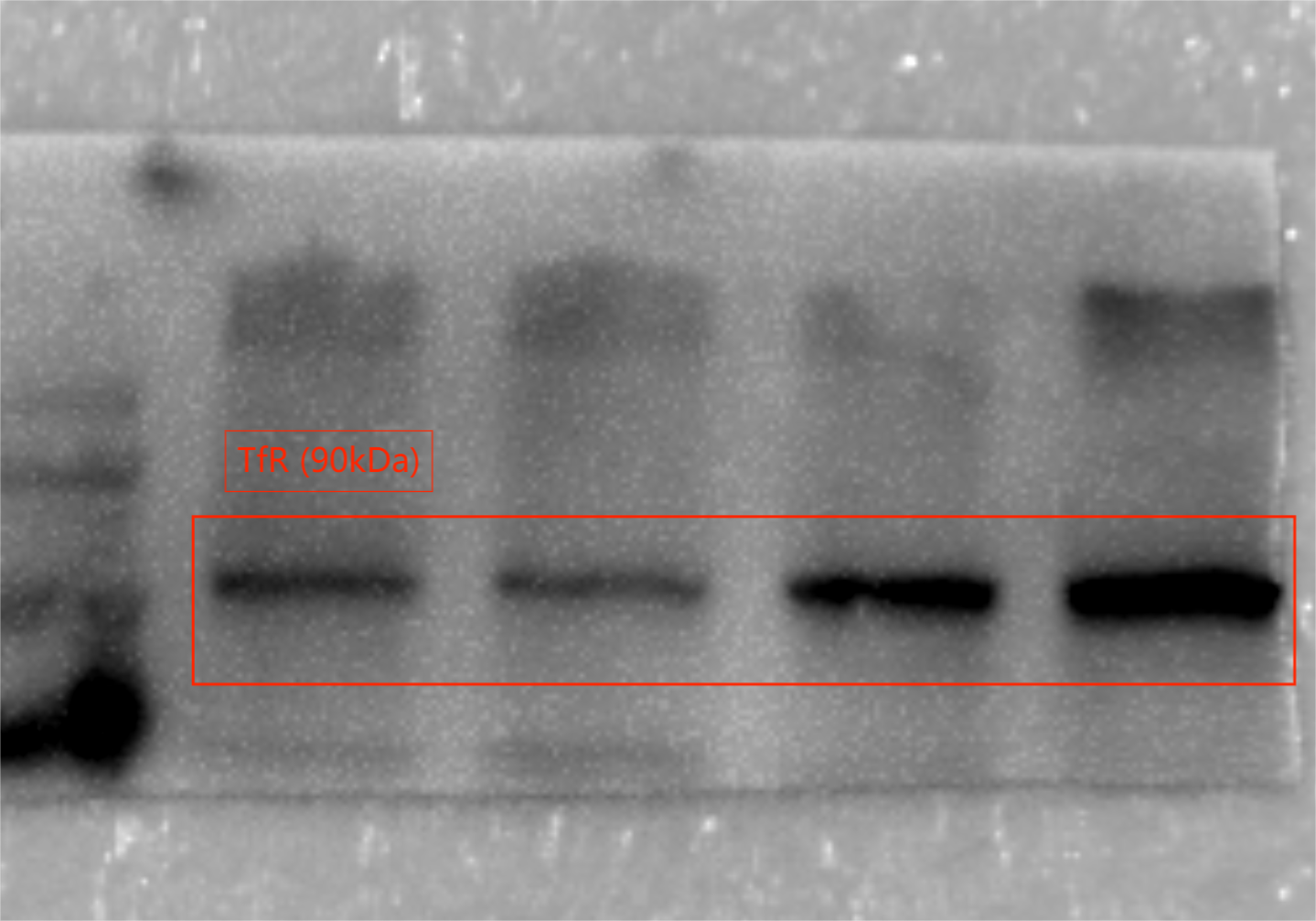

Supplement: Supplementary file 1 [file cancers-17-02349-s001.zip › WB/TfR.png]
